# Ligelizumab improves sleep interference and disease burden in patients with chronic spontaneous urticaria

**DOI:** 10.1002/clt2.12121

**Published:** 2022-02-16

**Authors:** Ana Giménez‐Arnau, Marcus Maurer, Jonathan Bernstein, Petra Staubach, Nathalie Barbier, Eva Hua, Thomas Severin, Yolandi Joubert, Reinhold Janocha, Maria‐Magdalena Balp

**Affiliations:** ^1^ Department of Dermatology Hospital del Mar Institut Mar d´Investigacions Mèdiques Universitat Autònoma Barcelona Spain; ^2^ Department of Dermatology and Allergy Dermatological Allergology Allergie‐Centrum‐Charité Charité ‐ Universitätsmedizin Berlin Berlin Germany; ^3^ Bernstein Allergy Group and Bernstein Clinical Research Center College of Medicine Cincinnati Ohio USA; ^4^ Department of Dermatology University Medical Center Mainz Mainz Germany; ^5^ Novartis Pharma AG Basel Switzerland; ^6^ China Novartis Institutes for Biomedical Research Co. Ltd Shanghai China

**Keywords:** daily functioning, quality of life, sleep interference

## Abstract

**Background:**

Chronic spontaneous urticaria (CSU) negatively impacts patients' sleep, thereby reducing health‐related quality of life (HRQoL). Half of patients with inadequately controlled CSU report sleep interference often or every night, which can lead to depression, anxiety, social, and work‐related problems.

**Methods:**

This randomized, double‐blind, placebo‐controlled Phase 2b core study (NCT02477332) included adult patients ≥18 years with moderate to severe CSU inadequately controlled with H_1_‐antihistamines. The current analysis includes patients randomized to receive ligelizumab 72 or 240 mg, omalizumab 300 mg or placebo every 4 weeks (q4w) for five injections over 20 weeks with treatment‐free follow‐up for 24 weeks. Patients could enter the open‐label extension study (NCT02649218) from Week 32 onwards if their weekly urticaria activity score was ≥12, which included an open‐label treatment (52 weeks of ligelizumab 240 mg q4w) and a 48‐week post‐treatment follow‐up. Weekly Sleep Interference Scores (SIS7, range 0 [no interference]–21 [substantial interference]), Weekly Activity Interference Score (AIS7), Dermatology Life Quality Index (DLQI) scores, and Overall Work Impairment were assessed.

**Results:**

Mean baseline SIS7 scores were balanced between the treatment arms for ligelizumab 72 mg (*n* = 84) and 240 mg (*n* = 85), omalizumab 300 mg (*n* = 85), and placebo (*n* = 43). By Week 12, patients experienced large improvements in sleep interference, with least square mean (standard error) changes from baseline (CFB) in SIS7 of −7.84 (0.58), −7.55 (0.61), −6.98 (0.60), and −5.85 (0.81), respectively. By Week 12, CFB in AIS7 were −8.25 (0.57), −8.25 (0.59), −7.30 (0.60), and −5.62 (0.79), DLQI scores were −9.79 (0.77), −9.93 (0.81), −8.35 (0.79), and −6.99 (1.11), and Overall Work Impairment scores were −28.96 (3.73), −30.76 (3.71), −25.74 (3.91), and −20.13 (5.10) for ligelizumab 72 and 240 mg, omalizumab 300 mg and placebo, respectively. Improvements in each patient‐reported outcome were sustained with ligelizumab 240 mg treatment during the extension study.

**Conclusions:**

Ligelizumab showed effective and sustained responses in managing sleep interference in patients with CSU, and numerically higher responses than with omalizumab and placebo. Treating the symptoms of CSU with ligelizumab improved disease burden, HRQoL, and markedly improved sleep quality.

## 1 BACKGROUND

The core manifestations of chronic spontaneous urticaria (CSU) present on the skin, however, its negative impact reaches far beyond, affecting patient's health‐related quality of life (HRQoL).^1^, ^2^, ^3^, ^4^, ^5^ Significant sleep interference is associated with CSU, which occurs at almost double the rate of that seen in age‐ and sex‐matched controls without CSU (58.0% vs. 32.7%, *p* < 0.001),^6^ and is also linked to CSU disease activity,^7^ being worse during urticaria flare‐ups.^8^ CSU disease state has been shown to have a direct and significant impact on sleep interference scores.^7^ An Internet survey of European patients with chronic urticaria (CU) revealed that, importantly, respondents were most commonly bothered by symptoms in the evening (34%) and at night (23%). The frequency of sleep affected was three nights per week, on average, and sleep disturbances from CU were inadequately addressed in 48% of patients, meaning it is a standard part of the condition.^9^ When compared with other dermatologic conditions, a significantly higher proportion of CU patients reported sleep difficulties in the past 12 months versus patients with any severity of psoriasis (40.8% for mild, 47.2% for moderate/severe psoriasis, and 55.7% for CU patients, *p* < 0.001).^10^ In patients with CSU, sleep is understudied, and sleep problems are generally not well understood. Possible contributing factors include difficulty in falling asleep and sleeping through the night because of the itch associated with hives.^11^, ^12^ Angioedema, which is present in up to 70% of patients with CSU, may also affect sleep.^13^ Wakefulness promoted by histamine released from mast cells and comorbidities such as sleep apnea^14^ may also contribute to sleep interference. It is clear that sleep loss negatively affects performance at work and daily functioning, significantly impacts social lives, and is associated with lower life expectancy.^15^ Even transient sleep loss can impair cognitive performance and judgment, and lead to an increased risk of other comorbidities, such as cardiovascular disease.^16^ Addressing the problem of sleep could have the potential to impact the overall health and wellbeing of patients with CSU.

Ligelizumab is a next‐generation, high‐affinity 97% (Novartis data on file) humanized monoclonal anti‐IgE antibody that has previously shown dose‐dependent and time‐dependent suppression of free IgE, the expression of its high affinity receptor, FcεRI, and skin‐prick test responses to an allergen, to a greater extent and duration than omalizumab.^17^ The interaction of ligelizumab with IgE has been shown to be around 88‐fold stronger than the IgE binding of omalizumab.^18^ In a randomized, Phase 2b core study (NCT02477332), the main objective was to determine a dose–response relationship for ligelizumab for the complete control of hives (indicated by a weekly hives‐severity score of 0) assessed at Week 12. Ligelizumab was compared to omalizumab and placebo in patients with CSU who were inadequately controlled with standard‐of‐care therapy including H_1_‐antihistamines (H_1_‐AH). Ligelizumab showed the most effective results by improving urticaria activity, reducing the signs and symptoms of hives, itch, and angioedema.^17^ Patients who presented with active disease after 32 weeks could enter an extension study to assess long‐term safety (primary endpoint) and long‐term efficacy (secondary endpoints).^19^

Here, we present a sub‐analysis from this Phase 2b core study and the extension study to evaluate sleep, activity interference, dermatology quality of life, and impact on work in patients with CSU treated with ligelizumab and omalizumab versus placebo.

## 2 METHODS

### 2.1 Study design and patients

**FIGURE 1:**
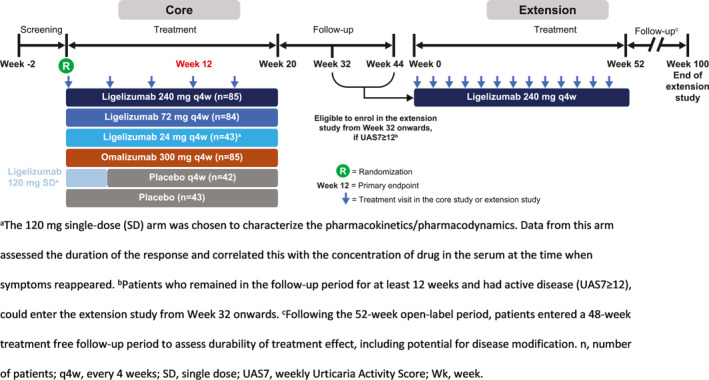
Study design of core and extension studies

The study design has been previously reported in detail.^17^ Briefly, in this randomized, double‐blind, active‐ and placebo‐controlled Phase 2b study, adult patients with moderate to severe CSU (defined by weekly urticaria activity score [UAS7] ≥16 on a scale from 0 to 42) were randomized in a 2:2:2:1 ratio to receive ligelizumab 72 or 240 mg, or omalizumab 300 mg, or placebo every 4 weeks (q4w) for five injections (see Figure 1 for core Phase 2b and extension study design). Patients also received ligelizumab 24 mg q4w or a single 120‐mg dose of ligelizumab, but these doses were out of scope and not included in our current analyses.

The extension study was an open‐label, single‐arm, long‐term safety Phase 2b extension for all patients who completed the Phase 2b core study and had a UAS7 ≥12 at Week 32.^19^ The extension study consisted of an open‐label treatment period (0–52 weeks of ligelizumab 240 mg q4w for 13 treatment cycles) and a treatment‐free follow‐up period until Week 100.^19^ The treatment‐free follow‐up period started 4 weeks after the last dose and continued for 48 weeks, with visits every 12 weeks, and assessed safety and long‐term treatment outcomes, including sustained remission.

In the current analysis, 297 patients were included in the Phase 2b core study as follows: ligelizumab 72 mg (*n* = 84), ligelizumab 240 mg (*n* = 85), omalizumab 300 mg (*n* = 85), and placebo (*n* = 43). Two additional arms included ligelizumab 24 mg q4w and a single dose of 120 mg (to characterize pharmacokinetics and pharmacodynamics); however, these treatment arms are not reported in the current analysis. In the extension study, 226 patients entered and were treated with ligelizumab 240 mg.

### 2.2 Assessment of patient‐reported outcomes

Each day during the entire study, patients completed the Urticaria Patient Daily Diary (UPDD), including the urticaria activity score (UAS), which assessed itch and hives, sleep interference (recorded every morning), and activity interference (recorded every evening).^20^, ^21^ Weekly scores were calculated as follows: UAS7 score ranging from 0 to 42, Weekly Sleep Interference Scores (SIS7), and Weekly Activity Interference Scores (AIS7) both ranging from 0 to 21.^20^, ^21^ Higher scores mean higher interference with sleep and activity, respectively.

Patients also recorded DLQI at baseline and every 4 weeks. DLQI has a recall period of 7 days and the total score ranges from 0 to 30 (higher scores reflect worse HRQoL).^22^, ^23^ The impact on work due to CSU was assessed using the Work Productivity and Activity Impairment‐Chronic Urticaria (WPAI‐CU version 2.0) with a 7‐day recall period.^24^ The six‐item WPAI‐CU has four subscales, including Absenteeism, Presenteeism, Overall Work Impairment (a composite score of Absenteeism and Presenteeism), and Activity Impairment, expressed as impairment percentage (0%–100%), with higher scores reflecting higher impairment and less productivity.

Wherever available, data from the Phase 2b core study are presented from Week 32; however, some endpoints were not measured at Week 32; therefore, we present data from Week 28 (DLQI and Overall Work Impairment).

### 2.3 Statistical analysis

In this analysis, we present change from baseline (CFB) data for patients across each treatment arm (ligelizumab 72 and 240 mg and omalizumab 300 mg) and placebo at various time points. The descriptive summary statistics are provided for the SIS7 scores and DLQI scores over time based on the observed data. Mixed models of repeated measures were performed on SIS7 scores, AIS7 scores, DLQI and Overall Work Impairment and Presenteeism with UAS7 responder status at each visit, and age, sex and duration of CSU as fixed effect factors with subject as a random effect using a compound symmetry covariance matrix.

Least square (LS) means are presented together with standards errors (SE) overtime for CFB. *p*‐Values provided are nominal. Week 12 Presenteeism and Overall Work Impairment was analyzed using Van‐Elteren test with background medication type as the stratification variable. No multiplicity adjustments were made, therefore, statistical interpretation should be made with caution.

## 3 RESULTS

### 3.1 Baseline characteristics from the Phase 2b core and extension studies were well balanced

Of the 382 patients who were randomly assigned to a treatment arm in the core study (including the 24 and 120 mg single ligelizumab), 338 patients (88%) completed the treatment phase of the study and all baseline demographics and clinical characteristics were balanced between the treatment arms (Table 1).^17^ In addition, baseline demographics in the core study and for the 226 patients who entered the extension study were similar.

**TABLE 1:** Demographic and clinical characteristics of patients with chronic spontaneous urticaria in the Phase 2b core study and the Phase 2b extension study

Characteristics	Ligelizumab 72 mg (*N* = 84)	Ligelizumab 240 mg (*N* = 85)	Omalizumab 300 mg (*N* = 85)	Placebo (*N* = 43)	Core study total (*N* = 382)	Extension study (*N* = 226)
Age (years)	44.3 ± 12.4	42.9 ± 10.5	41.8 ± 13.1	45.4 ± 11.2	43.3 ± 12.5	44.5 ± 12.7
Female sex no. (%)	61 (73)	67 (79)	66 (78)	31 (72)	286 (75)	170 (75)
Body mass index^a^	28.5 ± 7.1	27.9 ± 6.1	28.1 ± 6.4	27.4 ± 6.5	27.91 ± 6.5	28.8 ± 7.3
Race no. (%)^b^						
Native American	1 (1)	0	0	0	1 (0.3)	1 (0.4)
Asian	20 (24)	19 (22)	12 (14)	9 (21)	76 (20)	51 (23)
Black	2 (2)	0	4 (5)	0	8 (2)	3 (1)
White	57 (68)	65 (77)	67 (79)	31 (72)	283 (74)	163 (72)
Other	3 (3.6)	1 (1)	1 (1)	3 (7)	12 (3)	6 (3)
Time since diagnosis of chronic spontaneous urticaria (years)	3.9 ± 5.4	4.1 ± 5.6	5.1 ± 7.5	3.6 ± 3.5	4.3 ± 6.0	4.75 ± 6.2
IgE level IU/ml						
Median	101.0	74.1	86.2	111.5	87.2	104.5
Range	0–942.0	0–3480.0	0–14,100.0	2.2–870.0	0–14,100.0	0–2000
Weekly itch‐severity score^c^	13.6 ± 4.1	13.0 ± 4.3	12.7 ± 4.4	13.6 ± 4.1	13.1 ± 4.1	12.5 ± 4.9
Weekly hives‐severity score^c^	18.1 ± 4.3	17.3 ± 4.1	16.6 ± 4.7	17.6 ± 4.1	17.3 ± 4.4	15.7 ± 5.3
Weekly urticaria activity score^d^	31.7 ± 7.3	30.3 ± 7.3	29.3 ± 7.9	31.1 ± 6.8	30.4 ± 7.4	28.2 ± 9.1
Positive chronic urticaria index no. (%)^e^	32 (38)	35 (41)	33 (39)	14 (33)	145 (38)	81 (36)
Background medication						
Locally approved dose of H1‐antihistamine	35 (42)	36 (42)	37 (44)	19 (44)	164 (43)	102 (45)
Escalated dose of locally approved H1‐antihistamine	49 (58)	49 (58)	48 (56)	24 (56)	218 (57)	124 (55)

*Note*: Plus–minus values are means ± SD. There were no notable imbalances among the trial groups regarding the demographic and clinical characteristics of the patients at baseline.

^a^ The body‐mass index is the weight in kilograms divided by the square of the height in metres.

^b^ Race was reported by the patient or determined by the investigator.

^c^ The weekly itch‐severity and hives‐severity scores measure the severity of itch and hives, respectively, over a period of 7 days on scales ranging from 0 to 21, with higher scores indicating greater severity.

^d^ The weekly urticaria activity score is a composite of the weekly itch‐severity and hives‐severity scores. Scores range from 0 to 42, with higher scores indicating greater severity.

^e^ A positive Chronic Urticaria (CU) Index (scores range from 1 to 50, with scores ≥10 representing a positive result) indicates that the patient has either an autoimmune basis for the urticaria or an alternative histamine‐releasing factor that has been associated with greater disease severity than that in patients with a negative CU Index. The 120 mg single‐dose arm was chosen to characterize the pharmacokinetics/pharmacodynamics; the ligelizumab 120 and 24 mg arms are not included in the table but are included in the total column.

### 3.2 Many patients with CSU experienced sleep impairment at baseline, some of which was substantial

At baseline of the Phase 2b core study, the mean (standard deviation [SD]) SIS7 scores were balanced between treatment arms: 11.13 (5.57), 10.29 (6.00), 10.00 (5.51), and 10.91 (5.00), for ligelizumab 72 mg (*n* = 84) and 240 mg (*n* = 85), omalizumab 300 mg (*n* = 85), and placebo (*n* = 43) arms, respectively. At baseline, almost 12% of patients in each arm had a SIS7 score above 17. At baseline, the majority of patients had SIS7 between 3 and ≤17 for each treatment arm, with a rate of between 33.3% and 44.2%.

### 3.3 Ligelizumab achieved numerically greater improvements in Weekly Sleep Interference scores compared with placebo

**FIGURE 2:**
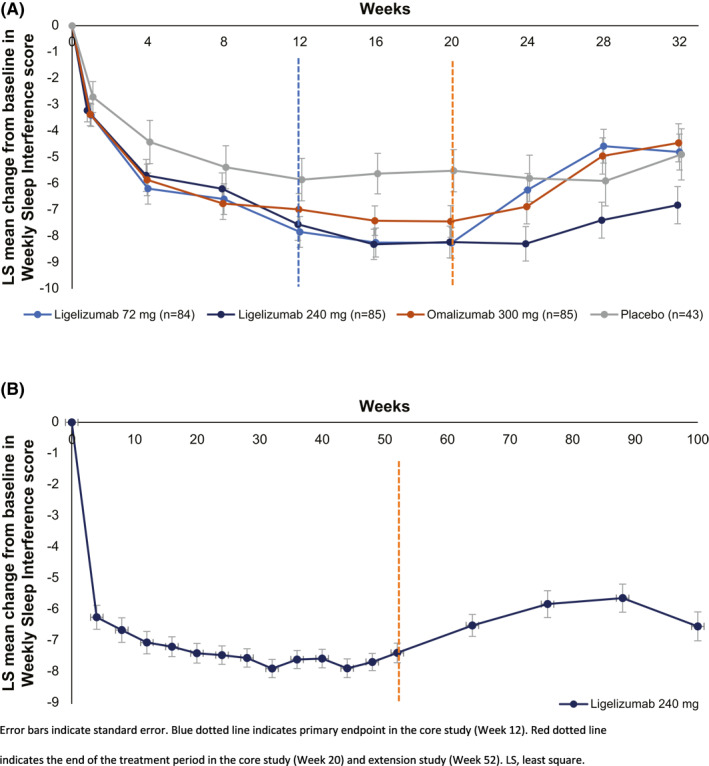
(A) LS mean change from baseline in Weekly Sleep Interference scores during the Phase 2b core study and treatment‐free follow‐up period and (B) the extension study and treatment‐free follow‐up period

By Weeks 12 and 20, patients had achieved numerically greater CFB responses in the active arms of the Phase 2b core study: the LS mean (standard error [SE]) CFB in SIS7 scores at Week 12 was −7.84 (0.58), −7.55 (0.61), −6.98 (0.60), and −5.85 (0.81), and by Week 20, it was −8.25 (0.58), −8.22 (0.60), −7.44 (0.60), and −5.51 (0.80) for ligelizumab 72 mg, 240 mg, omalizumab 300 mg and placebo, respectively (Figure 2A). By Week 20, 64.3%, 63.5%, 61.2%, and 51.2% of patients had no or limited sleep interference in the ligelizumab 72 and 240 mg, omalizumab 300 mg, and placebo arms, respectively. During the treatment‐free follow‐up period (from Week 20 up to Week 32), the effect on SIS7 was maintained and patients in the ligelizumab 240‐mg arm had a slower return to Baseline versus all the other arms (Figure 2A).

Patients who entered the extension study experienced sustained improvements in their sleep interference scores from baseline (−3.91 [0.31]) throughout the entire treatment period, starting from the first ligelizumab 240 mg dose at Week 0 until Week 52, with the greatest improvement observed at Week 32 (−7.87 [0.29]). Scores stayed low during the treatment period, with a gradual increase of SIS7 scores in the treatment‐free follow‐up period up until Week 100 (Figure 2B).

### 3.4 Weekly Activity Interference scores improved and remained stable throughout treatment with ligelizumab

**FIGURE 3:**
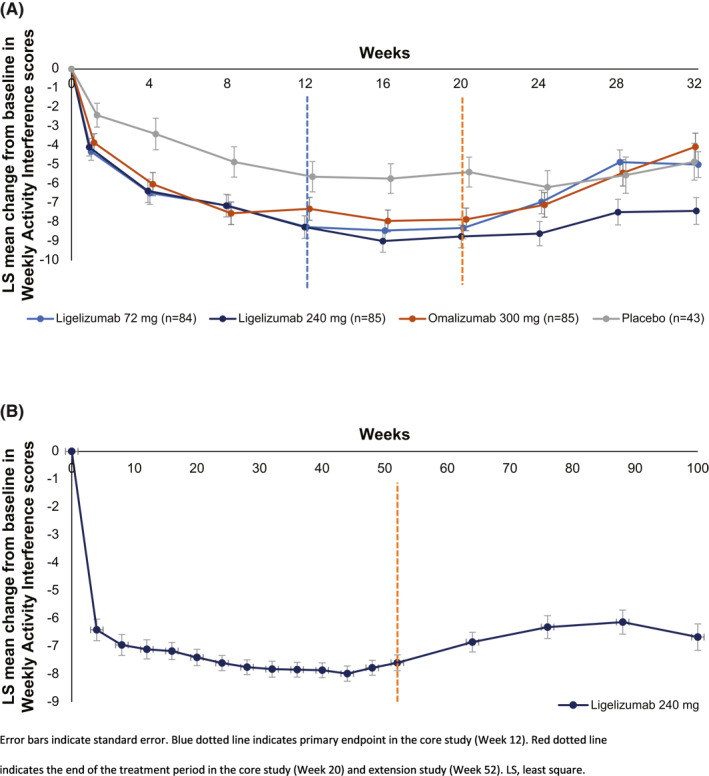
(A) LS mean change from baseline in Weekly Activity Interference score during the Phase 2b core study and treatment‐free follow‐up period and (B) the extension study and treatment‐free follow‐up period

By Week 12 of the Phase 2b core study, the LS mean (SE) CFB in AIS7 scores were −8.25 (0.57), −8.25 (0.59), −7.30 (0.60) and −5.62 (0.79), for ligelizumab 72 mg, 240 mg, omalizumab 300 mg, and placebo, respectively; by Week 20, AIS7 CFB remained stable at −8.30 (0.56), −8.74 (0.59), −7.85 (0.59), and −5.38 (0.78), respectively (Figure 3A). The improvement in AIS7 was sustained throughout the treatment period of the extension study (Figure 3B).

### 3.5 Dermatology Life Quality Index improvement was numerically greater for patients treated with ligelizumab compared with omalizumab and placebo

**FIGURE 4:**
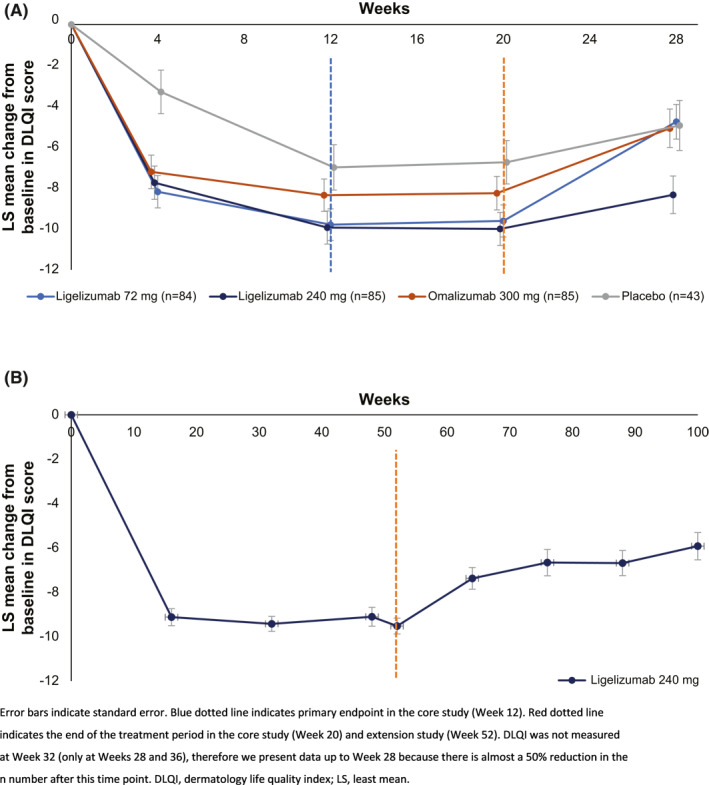
(A) LS mean change from baseline in Dermatology Life Quality Index score during the Phase 2b core study and treatment‐free follow‐up period and (B) the extension study and treatment‐free follow‐up period

The LS mean (SE) CFB in DLQI scores in the Phase 2b core study was numerically higher in the ligelizumab treatment arms at both Week 12 and Week 20: at Week 12, it was −9.79 (0.77), −9.93 (0.81), −8.35 (0.79), and −6.99 (1.11), and at Week 20, it was −9.61 (0.78), −10.0 (0.81), −8.25 (0.82), and −6.74 (1.06) for ligelizumab 72 and 240 mg, omalizumab 300 mg, and placebo, respectively (Figure 4A).

Patients in the extension study experienced sustained improvements in DLQI scores throughout the treatment period with the greatest improvement measured at Week 52 (−9.52 [0.36]). Improvements continued until Week 100 (−5.92 [0.62]) but were not as high during the treatment‐free follow‐up (Figure 4B).

### 3.6 Ligelizumab led to numerically greater improvements in Presenteeism and Overall Work Impairment compared with omalizumab and placebo

**FIGURE 5:**
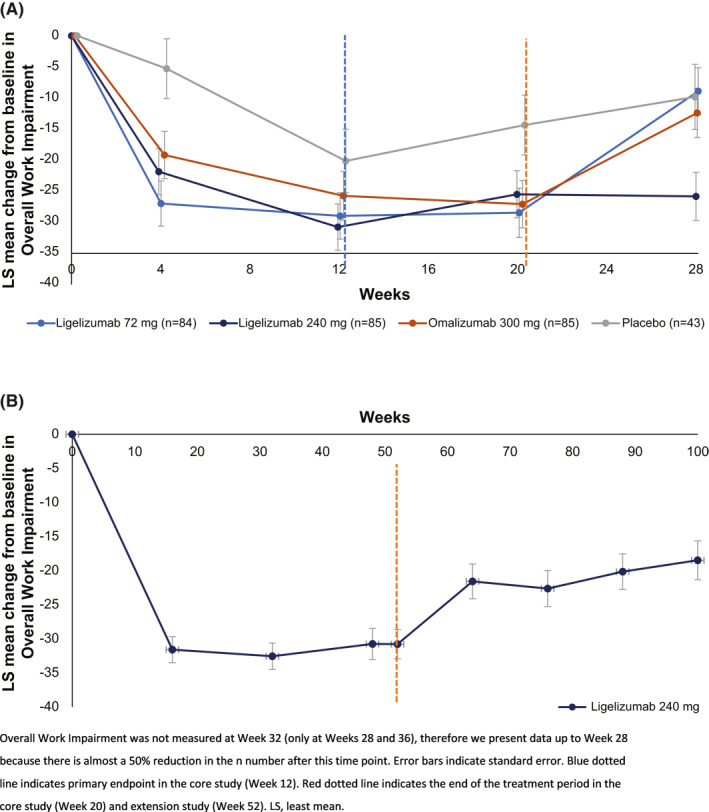
(A) LS mean change from baseline in Overall Work Impairment score during the Phase 2b core study and treatment‐free follow‐up period and (B) the extension study and treatment‐free follow‐up period

The LS mean CFB in Presenteeism and Overall Work Impairment was numerically higher in the ligelizumab treatment arms compared to omalizumab 300 mg and placebo (see Table 2 and Figure 5A). By Week 12 of the Phase 2b core study, patients on ligelizumab 240 mg achieved the highest improvement in Overall Work Impairment scores of −30.76 (3.71) versus −28.96 (3.73), −25.74 (3.91), and −20.13 (5.10) for ligelizumab 72 mg, omalizumab 300 mg, and placebo, respectively. At Week 12, Presenteeism had significantly improved for ligelizumab 72 mg (*p* = 0.004) and 240 mg (*p* = 0.003) versus placebo, and Overall Work Impairment significantly improved (*p* = 0.003 and *p* = 0.002, respectively) versus placebo; neither parameter improved significantly with omalizumab 300 mg treatment versus placebo. Improvements in Presenteeism and Overall Work Impairment were also experienced by patients in the extension study (Figure 5B).

**TABLE 2:** Absolute mean change from baseline in presenteeism

	Ligelizumab 72 mg (*N* = 84)	Ligelizumab 240 mg (*N* = 85)	Omalizumab 300 mg (*N* = 85)	Placebo (*N* = 43)
Phase 2b Core study				
Week 12, LS mean (SE)	−26.60 (3.43)	−28.99 (3.36)	−23.54 (3.53)	−17.54 (4.54)
Week 20, LS mean (SE)	−27.97 (3.61)	−26.76 (3.39)	−24.30 (3.45)	−13.36 (4.31)
Extension study				
Week 32, LS mean (SE)	NA	−35.33 (1.39)	NA	NA
Week 52, LS mean (SE)	NA	−33.63 (1.62)	NA	NA

*Note*: Week 12 is the primary endpoint in the Phase 2b core study and Week 20 is the end of the treatment period. Week 52 indicates the end of the treatment period in the extension study.

Abbreviations: LS, least square; *N*, number of patients; NA, not available; SE, standard error.

## 4 DISCUSSION

The analysis presented herein shows the correlations between CSU and various dimensions of HRQoL such as sleep and activity interference, dermatology‐QoL, and impact on work. The results suggest that treating the symptoms of CSU also has beneficial effects on these aspects of life. Treatment with ligelizumab, which improved urticaria activity, was associated with improvements in HRQoL (as reflected in DLQI sores) and sleep, as seen through reduced SIS7 scores. In addition, interference with daily activities was reduced (reflected by improvement in AIS7 scores) as well as a positive impact on work components (Presenteeism and Overall Work Impairment scores). These improvements with ligelizumab occurred as early as after the initial treatment (Week 4 of the core study, as shown in the figures), with continued improvements up to the end of the treatment period (Week 20) of the core study.

During the treatment‐free follow‐up period of the Phase 2b core study (Week 32), patients from the ligelizumab treatment arms maintained low SIS7 scores, with sleep interference relapse taking longer with ligelizumab 240 mg. Low SIS7 scores were maintained throughout treatment during the extension study. The mean DLQI scores continued to improve until the end of treatment in the Phase 2b core study and extension study, with the largest change observed in the ligelizumab 240 mg group. It has previously been shown that a DLQI score of between 3.3 and 4 relates to a minimal clinically important difference (MCID).^25^ DLQI scores for all arms in this study achieved an MCID after 12 weeks, but were higher for the ligelizumab arms: scores reduced (improved) by −9.79, −9.93, −8.35, and −6.99 and by Week 12, and −9.61, −10.0, −8.25, and −6.74 by Week 20 for ligelizumab 72 and 240 mg, omalizumab 300 mg, and placebo, respectively. In the extension study, the MICD in DLQI scores were sustained with ligelizumab 240 mg, with values of −9.52 at Week 52, and −5.92 at Week 100. The results presented from both the core and extension studies support previous findings of sleep interference and the relationship on work productivity and HRQoL.^1^, ^2^, ^3^, ^4^, ^5^ The effect of treatment on work activity was significant at Week 12 for both doses of ligelizumab; Presenteeism and Overall Work Impairment significantly improved for both ligelizumab doses versus placebo at Week 12; neither parameter improved significantly with omalizumab 300 mg treatment versus placebo. Mirroring the changes in other HRQoL parameters, the improvements in AIS7 scores were also notably reduced by Week 12 and 20, again with the largest change in the ligelizumab 240 mg arm by Week 20 (end of treatment period). Similar to the results seen in our study, the previous ASSURE trial showed that CSU markedly interferes with sleep and daily activities, and >20% of patients reported ≥1 h per week of missed work.^3^ All of these effects worsened with increasing disease activity.

Patients' responses to treatment can alter their sleep quality and levels of fatigue, and produce undesirable effects, as observed through the use of first‐generation H_1_‐AH. The global EAACI/GA^2^LEN/EDF/WAO (European Academy of Allergology and Clinical Immunology/Global Allergy and Asthma European Network/European Dermatology Forum/World Allergy Organization) guidelines^26^ on the management of urticaria are clear; they state that second‐generation, non‐sedating H_1_‐AH should be used for first‐line treatment of CSU, with a dosage increase up to fourfold as second‐line treatment. Comorbidities and the effect of H_1_‐AHs were not studied in our Phase 2b core or extension studies and warrant further investigation. Some patients experience daytime sedation as a consequence of using H_1_‐AH to control CSU symptoms. One of the objectives when choosing a treatment is to provide complete symptom control, including avoidance of sleep disturbances induced by the disease or treatments. Using appropriate biologic treatments, which are unlikely to interfere with the sleep cycle, should be considered in the right patients based upon a physician's diagnosis.

Sleep improvements have also been experienced in patients with CSU after treatment with omalizumab, as shown by the results from three randomized, placebo‐controlled trials.^27^ Sleep improvements were observed after the first dose of omalizumab, which mirrored the efficacy in disease activity, a positive effect that continued with subsequent doses, and improved throughout treatment. When discontinued, CSU symptoms returned along with rebounding sleep problems.^27^ Additionally, improvement in itch was associated with decreases in somnolence and sleep disturbances.

This study was limited by the relatively low number of patients and short trial duration of the core study, although the extension study showed longer‐term effects.^19^ Ligelizumab is being investigated in an ongoing clinical trial program that includes the Phase 3 PEARL 1 (NCT03580369) and PEARL 2 (NCT03580356) trials, which aim to recruit more than 2000 patients with CSU across 48 countries globally. The complex relationship between patients with CSU and sleep is imperative to study and these studies will help further our understanding and provide invaluable insights.

## 5 CONCLUSIONS

Overall, ligelizumab treatment has benefits beyond relief from urticaria symptoms and can impact HRQoL, highlighting the importance of targeting complete urticaria control. Treating the symptoms of CSU improves urticaria activity and HRQoL, and thereby also has the potential to improve sleep (through decreased interference),^28^ which should be confirmed in further Phase 3 studies. Sleep interference is drastically underappreciated as a severe and clinically relevant symptom of CSU. Patient assessments of the effect of therapy on sleep quality should explicitly be included as part of an integrated treatment approach.

## CONFLICT OF INTEREST

Ana Giménez‐Arnau reports roles as a Medical Advisor for Uriach Pharma, Sanofi and Genentech, Novartis, FAES, GSK, Thermo Fisher, Almirall, Amgen, and has research grants supported by Uriach Pharma, Novartis, and Instituto Carlos III‐ FEDER; she also participates in educational activities for Uriach Pharma, Novartis, Genentech, Menarini, LEO‐PHARMA, GSK, MSD, Almirall, Avene, and Sanofi. Marcus Maurer is or recently was a speaker and/or advisor for and/or has received research funding from Allakos, Amgen, Aralez, ArgenX, AstraZeneca, Celldex, Centogene, CSL Behring, FAES, Genentech, GIInnovation, Innate Pharma, Kyowa Kirin, Leo Pharma, Lilly, Menarini, Moxie, Novartis, Roche, Sanofi/Regeneron, Third HarmonicBio, UCB, and Uriach. Jonathan Bernstein reports grants and personal fees from Celldex, Amgen, Sanofi Regeneron, Novartis, Astra Zeneca, Allakos, and Genentech. Petra Staubach has received research funding and/or fees for consulting and/or lectures from Novartis, CSL Behring, Shire, MSD, Schering‐Plough, Abbvie, Viropharma, Leo Pharma, Leti Pharma, Pohl‐Boskamp GmbH, Astella, Allergika, Karrer, Allmirall, Sanofi, Octapharma, Pfleger GmbH, Beiersdorf, L'Oreal, Lilly, Janssen, Celgene, Hermal, UCB, Allmirall, Astelas, Sobi, and Pfizer. Eva Hua is an employee of China Novartis Institutes for Biomedical Research Co. Ltd, China. Nathalie Barbier, Thomas Severin, Yolandi Joubert, and Maria Magdalena‐Balp are full‐time employees of Novartis Pharma AG, Basel, Switzerland. Reinhold Janocha is a former Novartis employee and holds Novartis shares.

## AUTHOR CONTRIBUTIONS

**Ana Giménez‐Arnau:** Conceptualization; Data curation; Formal analysis; Investigation; Supervision; Validation; Writing ‐ review & editing. **Marcus Maurer:** Conceptualization; Formal analysis; Investigation; Supervision; Validation; Writing ‐ review & editing. **Jonathan Bernstein:** Investigation; Supervision; Validation; Writing ‐ review & editing. **Petra Staubach:** Investigation; Validation; Writing ‐ review & editing. **Nathalie Barbier:** Data curation; Formal analysis; Methodology; Project administration; Resources; Validation; Writing ‐ review & editing. **Eva Hua:** Data curation; Formal analysis; Methodology; Resources; Supervision; Validation; Writing ‐ review & editing. **Thomas Severin:** Data curation; Formal analysis; Funding acquisition; Methodology; Project administration; Resources; Supervision; Validation; Writing ‐ review & editing. **Yolandi Joubert:** Data curation; Formal analysis; Methodology; Supervision; Validation; Writing ‐ review & editing. **Reinhold Janocha:** Data curation; Formal analysis; Methodology; Validation; Writing ‐ review & editing. **Maria‐Magdalena:** Conceptualization; Data curation; Formal analysis; Methodology; Supervision; Validation; Writing ‐ review & editing.
